# Radiologists’ experiences and perceptions regarding the use of teleradiology in South Africa

**DOI:** 10.4102/sajr.v27i1.2647

**Published:** 2023-08-31

**Authors:** Renata Schoeman, Mario Haines

**Affiliations:** 1 Department of Leadership, University of Stellenbosch Business School, Bellville, South Africa; 2 Department of Healthcare Leadership, Faculty of Economic and Management Sciences, University of Stellenbosch Business School, Bellville, South Africa; 3 Diagnostic Radiologist, Private Practice, Pietermaritzburg, KwaZulu-Natal, South Africa

**Keywords:** teleradiology, benefits, challenges, opportunities, barriers, impact, radiologists, perceptions, LMIC, South Africa

## Abstract

**Background:**

Teleradiology was implemented in South Africa in 1999, but the subsequent uptake was low and slow. The onset of the coronavirus disease 2019 (COVID-19) pandemic catapulted South African healthcare into the arena of teleradiology. This created the environment for re-examining the factors that enable or inhibit the uptake of teleradiology in both the public and private sectors.

**Objectives:**

This article reports on a study of a select sample of private and public sector radiologists’ experiences with, and perceptions of, the benefits, opportunities, challenges and barriers to the implementation of teleradiology in the South African context.

**Method:**

Qualitative data on the perceived benefits and challenges of teleradiology, as well as on its enablers and the barriers to its implementation, were collected and analysed.

**Results:**

The uptake of teleradiology in the sample increased by 15.9% during the COVID-19 pandemic. The results demonstrated that teleradiology was perceived to have clear benefits on operational, personal and societal levels.

**Conclusion:**

It is important to address structural barriers to the implementation of teleradiology. Clear communication strategies and multistakeholder engagement are also required.

**Contribution:**

By investigating radiologists’ experience with teleradiology, this study provides an understanding of the benefits, opportunities, challenges and barriers to implementation of services. These insights enable informed decision-making and stakeholder engagement and provide a foundation for establishing recommendations for the viable implementation of teleradiology in South Africa and other lower- and middle-income countries to promote access to healthcare.

## Introduction

The World Health Organization (WHO) estimates that two-thirds of the world’s population experiences barriers to medical imaging access.^1^ Mendel et al.^2^ indicated that this lack of diagnostic radiology contributes to higher morbidity and mortality rates in lower- and middle-income countries (LMICs). South Africa’s dual healthcare system comprising public and private healthcare sectors is an example of a LMIC context, displaying glaring inequities in accessibility to and provision of services. In 2022, the Radiology Society of South Africa (RSSA) reported that 650 registered radiologists served a population of 59 million, of which the minority were based in the public health sector, which services 84% of South Africa’s population.^3^ The plight of individuals in remote, poorly resourced areas is aggravated as they often do not have the financial means to travel to distant health facilities to seek treatment.^4^

The European Society of Radiology defines teleradiology as ‘the exchange of radiological images and patient-related data between geographically different locations for primary interpretation, expert consultation and clinical review by digital transmission’.^5^ Teleradiology could therefore add value to early diagnosis and disease management in low-income, high disease-burdened countries such as South Africa. Hanna et al.^6^ assert that teleradiology could help mitigate discrepancies in healthcare by reducing access barriers and increasing imaging efficiency. Teleradiology has the potential to bridge the gap in remote and poorly resourced areas, improving the quality of medical care, reducing in-patient hospital stays, reducing treatment costs, enabling healthcare to use information and communication technologies cost effectively and securely, improving the availability, quality and use of information, and increasing access to healthcare services by reducing distance and time barriers.^7^

Early diagnosis and treatment can significantly improve patient outcomes.^8^ Two of the main drivers for the uptake of teleradiology are limited geographic reach owing to the remoteness of areas and a lack of skilled human resources.^9^ Both these factors prevail in the context of South Africa’s public health sector. However, teleradiology has not yet been embraced in the public sector – which needs it the most – despite being implemented in South Africa in 1999. In contrast, the private health sector has used teleradiology to assist with after-hours reporting from multiple sites by a centralised or single private healthcare sector.^10^ This led to the National Department of Health adopting telemedicine strategies to scale up healthcare service provision at district level.^11^

The coronavirus disease 2019 (COVID-19) pandemic catapulted South African healthcare into the arena of teleradiology. During South Africa’s state of emergency and the ensuing lockdown, patients were afraid to go to crowded and infected healthcare facilities, and consequently demand for radiological services dropped. However, teleradiology was used to preserve scarce resources for COVID-19 patients, take healthcare to the people and protect healthcare workers and patients from contracting the virus.^12,13^ The pandemic created an environment for re-examining the factors that enabled or inhibited the uptake of teleradiology in both the public and private sectors.

Previous research identified the following to be the most pertinent factors inhibiting the implementation of teleradiology: a lack of infrastructure (namely, picture archiving, communications systems and advanced hardware), a lack of radiology support, insufficient space, limited image storage capacity, ageing of systems, vendor-related concerns and financial constraints.^14^ A further restraint identified was unpreparedness for changes in how the healthcare team would work, with Essop and Kekana^15^ recommending upskilling individual end-users to bridge the knowledge gap to complement the extended roles in the teleradiology setting. The insights from this target group of end-users (i.e., radiologists) warrant further exploration.

The aim of this article is to report on radiologists’ experiences with, and perceptions of the benefits, opportunities, challenges and barriers to the implementation of teleradiology in the South African context during the COVID-19 pandemic. Exploring and understanding these factors provide a foundation for establishing recommendations for the viable implementation of teleradiology in South Africa and other LMICs as part of a solution to improve access to healthcare.

## Research methods and design

This article reports on a survey exploring radiologists’ experience with teleradiology and their perceptions of the value of teleradiology. This was a qualitative study with an inductive focus, aiming to make the tacit knowledge of the radiologists explicit. An online electronic survey was used to collect and understand the experiences and perceptions of radiologists about remote reporting. An invitation to participate accompanied the survey, which was distributed by the RSSA to all radiologists registered with the Health Professionals’ Council of South Africa (HPCSA) who were members of the RSSA (583 at the time of the study). Of these, 448 radiologists were employed in private practice and 127 in the public sector, while eight were employed in both the sectors.

The survey was based on the key issues identified during a review of the literature. The survey consisted of multiple-choice and open-ended questions. Demographic details such as age, gender, years since qualification, work environment and experience with teleradiology were recorded. Perceptions about benefits and challenges regarding quality, efficiency, productivity, collaboration and cost–benefits were also gathered and rated.

Participants were asked to provide an indication of their experience with (or the lack thereof) and their views on enablers of and barriers to the implementation of teleradiology, and to provide recommendations for the successful implementation of teleradiology in South Africa in open-ended questions. These responses were analysed using ATLAS.ti (version 9) for the qualitative thematic analysis of participants’ responses to the perceived role, benefits and disadvantages of teleradiology, as well as the perceived enablers and barriers to implementation. The process of Braun et al.^16^ was followed. All responses were coded and themes identified.

### Ethical considerations

Ethical approval was obtained in advance from the Departmental Ethics Committee of the University of Stellenbosch Business School (project no. 25887) and the RSSA. Eligible persons were invited to participate in the study via an email containing a link to the electronic survey. A formal agreement to participate was required before participants could complete the survey. The researcher had no access to personal identifiers as the invitation to participate in the survey was distributed by the RSSA. All responses were recorded anonymously in a web-based response set.

## Results

### Participants

Eighty-two responses were received (response rate 14.07%). Most of the sample (52; 63.4%) were between the ages of 30 and 50 years, while 51 (62.2%) identified as male. Radiologists represented seven provinces, with the majority practising in KwaZulu-Natal (*n* = 33; 40.2%), Gauteng (*n* = 25; 30.5%) and the Western Cape (*n* = 16; 19.50%). Thirty-three (40.2%) of the radiologists who participated had held their qualification for more than 15 years, while the second most represented group comprised those practising for 5–10 years (*n* = 23; 28%). Fifty-four (65.9%) participants were practising in the private sector, while only 12 (14.6%) were employed in the public sector. The remainder of the participants held either dual academic–private appointments or dual public–private appointments.

Prior to the COVID-19 pandemic, 52 participants (63.4%) employed remote access workstations (i.e., teleradiology). This number increased to 65 (79.3%) during the pandemic. Of the radiologists engaging in teleradiology, 42 (51.2%) indicated that their employers did not cover the costs associated with the technology of this remote mode of work.

### Qualitative analysis

During the thematic analysis, 858 quotations were extracted from the responses of the 82 participants to open-ended questions. Twenty-nine codes were used to label the benefits and limitations of teleradiology, as well as enablers of and barriers to implementation. These codes were clustered into six categories, namely benefits of teleradiology (160 quotes), limitations of teleradiology (124 quotes), enablers of implementation (106 quotes), barriers to implementation (205 quotes), impact on healthcare (106 quotes) and recommendations for implementation (157 quotes). From these categories, four themes emerged: operational impact, personal impact, societal impact and requirements for implementation. The quotations are referred to by participant number and quote number (e.g. P18, Q124).

#### Operational impact

Participants emphasised the positive impact of teleradiology on *productivity and efficiency*. Previously, radiographers captured photographs with digital cameras and then transferred them to a computer before sending to the radiologist. This laborious procedure involved internet-based file transmission to a radiologist for a diagnostic report. The introduction of modern technology simplified the process. Telecommunications facilitated instantaneous information flow, enhancing the efficiency of diagnostic reporting. Participants expressed the opinion that teleradiology enabled quicker diagnoses and reduced backlogs:

‘Greater throughput and less backlog of work, as the work can be distributed over a greater pool of radiologists.’ (P17, Q157)

‘Overall, will result in more efficient practice and reduced costs if smaller hospitals have scanning facilities. No need for transfers, reduced delays in diagnosis and prevention of complications.’ (P21, Q195)

Teleradiology could circumvent or minimise the unavoidable interruptions that radiologists face at healthcare facilities, for example, noise and constant foot traffic of other care workers. One participant reported that it:

‘[*G*]ives you the opportunity to report in a controlled environment with no distractions, allowing greater efficiency.’ (P45, Q419)

*Collaboration enhances quality of care* was identified as having operational impact. Skilled and trustworthy physicians are essential for quick and accurate diagnoses. Teleradiology facilitates access to facilities and experts across the world for imaging, interpretation and diagnosis:

‘Teleradiology enhances the level of patient care and support, because it allows radiologists to extend their expertise to patients and physicians without having to be physically there with them.’ (P27, Q250)

‘Consultation for complex cases would be useful.’ (P37, Q345)

Furthermore, respondents noticed that as teleradiology is not time- and space-bound, radiology services can be available 24/7 and optimised, as one after-hours teleradiologist can stay optimally engaged, covering services across various locations and time zones (P36, Q334), while other colleagues can rest in readiness for the next shift. Even in situations with a reduced in-house complement of healthcare providers, the facility could maintain the level of service, supplementing its human resources with practitioners reporting remotely.

Apart from the sharing of human resources (P17, Q156), respondents also highlighted the *cost benefit* of boundless access to other facilities’ resources, which reduce infrastructure costs and operational costs (P27, Q247). Participants also suggested that South Africa can no longer afford the hidden costs of inefficiency (delayed diagnosis, mortality, extended hospital stays and unnecessary transfers between facilities for special investigations). In their opinion, the cost of technological innovation is worth it because it saves lives, improves service delivery and achieves the Sustainable Development Goals to which South Africa is committed:

‘Long-run reduce cost of healthcare as decisions to treat patient from remote location are made more effectively.’ (P1, Q3)

‘Cheap method of significantly broadening radiological access throughout South Africa that can be rapidly implemented.’ (P44, Q416)

Another advantage, especially in light of the COVID-19 pandemic, is the reduced *infection control* risk. By presenting healthcare facilities with less person-to-person contact, the spread of infectious diseases among patients and practitioners can be reduced in the workplace (P8, Q71).

However, teleradiology poses *communication and expectation challenges.* Access to crucial clinical and collateral information is often difficult. When a clinician refers a patient for specialist attention, it is imperative that the radiologist understands the prognosis or diagnosis. Having access to files and referring doctors assists the radiologist to help the patient. A lack of communication between clinicians, radiologists and referring doctors could be potentially detrimental to the diagnosis or intervention implemented (P46, Q433; P17, Q159; P65, Q613). Teleradiology may also pose challenges in managing expectations of those clinicians who insist on having radiologists physically present (P18, Q169). It is important that the relationship between clinicians, on-site radiologists and teleradiologists is preserved:

‘Radiologists will veer even further from the clinical closeness.’ (P50, Q472)

‘Should there be no onsite radiologist at the larger hospitals, the clinicians – particularly other specialists – might lose value for the onsite radiologists. The clinician may feel that the radiologist is too comfortable and takes too little clinical responsibility, which is already the view expressed by some clinicians. This may result in deterioration [*of*] the relationship between clinicians and radiologists.’ (P18, Q162)

‘Extra effort needed to discuss cases with colleagues or get a second opinion. Easy to ask the colleague in the practice with you.’ (P17, Q159)

Some participants considered face-to-face communication crucial to alleviate the stress experienced by line managers who need to coordinate departmental workflow and performance management (P36, Q388).

#### Personal impact

Participants welcomed the *flexibility and autonomy* that teleradiology affords. The nature of radiology facilitates off-site and after-hours reporting as it does not require the physical presence of the radiologist at the healthcare facility where the patient is located. Participants noticed a better work–life balance, reduced travel time and costs, and the financial savings teleradiology affords (P7, Q62; P77, Q726; P1, Q3; P14, Q128).

Participants observed their *personal growth* as teleradiology had increased their learning opportunities by exposing radiologists to global experts and a myriad of information (P59, Q551).

Participants welcomed the potential savings and *financial opportunities* such as the additional job and locum opportunities, over-time payments and being on standby for second opinion diagnoses that teleradiology creates (P77, Q726; P72, Q676). This might be offset against the ‘need to reduce your rates for remote consulting’ (P26, Q220). Some radiologists did express job security concerns, cautioning that the introduction of teleradiology should not replace resident radiologists (P17, Q162).

*Social isolation* and limited interaction with colleagues have a negative impact on well-being. One participant reported that ‘one might argue that online meetings could address this; however, online meetings are quite exhausting as they tend to be many and feel like they are never-ending’ (P14, Q132).

*Environmental constraints* in working from home or remotely need to be addressed. Distractions in the home environment, unstable internet connections and power outages may interrupt and delay work (P52, Q500; P71, Q672). Participants also commented on the inability to perform the ultrasound examination or to direct the imaging personally (P33, Q309; P36, Q338). The lack of on-site information technology support can also cause additional delays and frustrations. One participant observed the advantage of being close to scanners and radiographers ‘to give advice and help, where necessary, to obtain the best possible imaging’ (P81, Q768).

#### Societal impact

*Increased access to care* was seen as a potential benefit of teleradiology. Participants agreed that teleradiology should be widely adopted in South Africa to enhance the country’s healthcare delivery (P2, Q12; P12, Q117; P17, Q162). Teleradiology would help to ensure that all patients from both urban and rural backgrounds had access to high-quality radiology services:

‘For improved healthcare service to our country and to reduce burden to public healthcare.’ (P78, Q742)

‘Provide radiological services to under-resourced regions where image acquisition is possible but no qualified radiologist to report.’ (P38, Q354)

However, there is a caveat: to improve access to radiology in the public sector, willing, capable personnel and functional equipment are required (P31, Q288).

Participants had varying views of the impact of teleradiology on the *quality of care.* Worldwide, demand has grown for faster radiology diagnostic and image interpretation services. Participants felt that teleradiology allows healthcare to respond more promptly and efficiently to patients’ needs (P27, Q249). They expressed the opinion that teleradiology raises the level of patient care by promoting quicker decision-making about diagnoses and treatment (P27, Q249; P12, Q110).

Participants also suggested that teleradiology would enhance the skills of and networking between public- and private-sector radiologists and between local and foreign radiologists. Teleradiology will also self-facilitate the culture shift necessary for widespread implementation (P2, Q19). It is believed that this would raise the quality of care in the country and help South African radiologists to become more proactive and productive (P46, Q429).

However, some participants were concerned about possible compromised patient healthcare owing to delayed diagnosis and treatment or misdiagnosis because of poor imaging quality:

‘Lack of immediacy. Some if not most studies are reported well after the patient has long left the exam room and thus no modification in views can be made. Serial studies may preferably require presence of the reporting radiologist. Lots of time spent on the telephone.’ (P43, Q403)

‘Being off site can decrease imaging quality if not directly involved. Clinicians prefer in person contact. Immediate intervention not possible if off site.’ (P55, Q518)

Teleradiology has been widely criticised for the exorbitant setup costs. However, participants do acknowledge that the initial investment pays off in the long run, with cost savings to the facility and the practitioners, which increase the *affordability* of radiology services (P1, Q4; P63, Q595). Moreover, teleradiology offers a cost-effective and sustainable opportunity to address the lack of speciality knowledge, human resources, technology and general infrastructure of South African rural healthcare facilities (P2, Q13). However, the benefits must be weighed against the costs of perceived displacement or unemployment of resident radiologists (P17, Q162).

#### Requirements for implementation

Good *infrastructure* is a prerequisite for implementing teleradiology. Most participants cited a lack of financial support as a potential barrier to expanding teleradiology services in South African healthcare. Poor infrastructure, particularly the lack of reliable peripheral workstations in smaller, remote, less-equipped facilities in the public sector, is a possible barrier to the successful implementation of teleradiology (P13, Q124). Participants stressed the importance of investing in the expensive teleradiology technologies, such as picture archiving and communication systems (P58, Q547; P77, Q737). Worldwide, these technologies have transformed how patients are cared for, how medical research is performed, how doctors learn and how healthcare is administered. Effective and readily available information technology support is also needed for the successful implementation of teleradiology (P28, Q261).

Teleradiology involves the transmission of medical images between facilities. Inadequate and unstable *connectivity*, aggravated by regular power outages, presents a threat to the successful adoption of teleradiology in the South African healthcare system (P3, Q26; P41, Q386).

*Governmental support and policies* facilitating the implementation of teleradiology is necessary. Participants raised the lack of insight, poor decision-making and the negative impact of government corruption on healthcare generally and teleradiology specifically. These factors may negatively affect funding decisions by government relating to the rollout of cutting-edge teleradiology infrastructure (P1, Q8; P38, G358). A call for public–private partnerships was made to provide universal internet access to all South Africans and to lower data costs (P44, Q411).

Successful implementation of teleradiology requires *change management.* Although teleradiology is a technological system, the interaction between people and the technology plays a crucial role in achieving innovative healthcare. Participants raised concerns regarding the negative attitude of key opinion leaders in the radiology profession, which is perceived to stem from a lack of information, lack of training on the system, fear of change and distrust of the system (P46, Q434). Most participants alluded to the need for inclusion of radiologists in the decision-making process, a willingness to embrace change and consensus among healthcare stakeholders about the need to implement teleradiology:

‘Radiologists need to be part of decision-making teams for the radiology department rather than have people who are clueless about the department sit in decision-making committees.’ (P32, Q298)

## Discussion

Although South Africa’s uptake of teleradiology prior to the COVID-19 pandemic was low and slow, there was a 15.9% increase in remote telereporting since the onset of the pandemic. Ntja et al.^14^ ascribed the slow adoption of teleradiology to a lack of infrastructure, insufficient support, digital and technological challenges, vendor concerns, and financial constraints. However, the digital healthcare revolution in combination with the needs created by the pandemic (e.g., infection control requirements, and the increased disparity in accessibility of care between rural and urban areas) catapulted South Africa forward. Teleradiology was considered by participants to improve efficiency and quality of work, work–life balance and, ultimately, job satisfaction of radiologists. This agrees with Kwee and Kwee^17^ and Hannah et al.^6^ who, during the COVID-19 pandemic, highlighted the opportunities (and challenges) associated with the new remote work paradigm.

Teleradiology has evolved in step with developments in the healthcare environment, including changes in technology and expectations of patients and clinicians.^18^ The research participants considered that teleradiology had the potential to positively affect operational, personal and societal functionings. The findings indicated that teleradiology would improve the overall value of radiology services by increasing quality and decreasing costs. Quality of care for patients could be improved through collaboration among healthcare providers, increased access to healthcare (despite local resource constraints) and improved outcomes. Quality of healthcare professionals could be improved through personal growth opportunities and well-being. Although the initial investment in infrastructure and connectivity may be high, the affordability over time – owing to increased efficiency and productivity and reduced human and technology resource costs – will lead to ultimate cost savings.

The findings of this study concur with earlier studies, which highlighted the potential of teleradiology to bridge the gap between the demand for and availability of diagnostic services. Teleradiology provides specialised medical services to diverse geographic locations, addressing the shortage of radiologists and facilitating the sharing of specialist expertise and equipment between adequately equipped hospitals and those with resource constraints.^7,19^

Despite the clear potential that teleradiology has on operational, personal and societal levels, there are some risks that need mitigation. The important role of effective communication in delivering quality services, relationship building and expectation management is crucial, as previously highlighted by Abdulwahab^20^ and Goelz et al.^21^ Radiologists should educate patients and collaborating doctors that teleradiology is a valuable and helpful complement to on-site radiology services rather than a substitute for it. Virtual consultation with colleagues will significantly improve communication in teleradiology settings, allowing the referring clinician and reporting radiologist to communicate via a real-time computer screen share system. Both parties can view the images, control the display and point to interesting pathology while texting digitally or talking on the phone.

It is also important that the necessary support is in place to manage environmental challenges – both at the individual service provider level (e.g., the setup of suitable remote workstations) and on a broader South African level (e.g., contingency plans for power outages and more reliable and affordable connectivity options). The quality of teleradiology reporting should also be improved by developing formal teleradiology training, where radiographic technologists are also included in addition to, as proposed by Krupinski^18^, an obligatory quality assurance system. The South African eHealth strategy document lists universities in KwaZulu-Natal, Eastern Cape and the Western Cape that offer introductory telemedicine training programmes to develop competent and skilled telemedicine practitioners, which should include training for end-users of the system. However, the current courses cater for medical practitioners in the context of telemedicine and not teleradiology *per se*.^22^

### Strengths and limitations

This is the first study to explore the views of a large group of radiologists on teleradiology in South Africa as an example of a LMIC setting. The findings highlight the positive impact that teleradiology has on personal, organisational and societal levels – as highlighted by the COVID-19 pandemic. It also warns about the structural and human barriers to the adaptation of teleradiology, which emphasises the need for effective communication, training, and stakeholder engagement, and the need for supportive policies and funding.

This study was exploratory in nature, and although it would have been ideal to include all radiologists in South Africa in the study, access to contact details is challenging and the researchers opted to reach them via a representative body, rather than social media. Many radiologists are not members of the RSSA. The radiologists who participated in the study were predominantly from urban areas and the majority represented the private healthcare sector. This may have biased the results and overvalued and underestimated some of the benefits and challenges about teleradiology and the implementation thereof.

### Recommendations

Teleradiology can add value to early diagnosis and disease management, with improved health outcomes, in low-income, high disease-burdened countries. Given the findings of this study, the researchers recommend multilevel change management interventions to facilitate the successful implementation of teleradiology, thus unlocking its benefits.

At a societal level, a dedicated taskforce from the RSSA should draft a proposal or white paper for appropriate governance and effective policy development on a governmental level to support the capacitation of healthcare facilities and training of healthcare workers in the implementation of teleradiology. Strategic public–private partnerships should be encouraged for infrastructure development and improved connectivity.

On an operational level, upskilling of the workforce (radiographers, radiologists and information technology support) through dedicated programmes, as well as continuous professional development is needed. Specific upskilling regarding the use of technology for communication and service delivery is crucial. Formal change management processes may be required to address uncertainties, reluctance among healthcare practitioners and their lack of confidence to utilise teleradiology.

Teleradiology could raise the service level in the public healthcare sector, which attracts and employs few of South Africa’s radiologists. On an individual level, teleradiology mitigates human resource barriers to care through removing the need for relocation, poor work satisfaction from working in an under-resourced facility, lower salaries in the public sector and fewer opportunities for career advancement. Therefore, the public sector and remote geographic facilities could attract more expertise from remote reporting clinicians.

## Conclusion

This study investigated the experiences of a select sample, albeit a relatively large group, of radiologists with teleradiology. The uptake of teleradiology increased during the COVID-19 pandemic, which prompted the investigation into widespread implementation of teleradiology in South Africa. Understanding the benefits, opportunities, challenges and barriers of implementation of these services enables informed decision making and stakeholder engagement to reap the societal (access to quality healthcare), operational (efficiency and quality) and personal (flexibility, well-being and growth) benefits of teleradiology.
